# The move towards living systematic reviews and living guidelines in healthcare: consideration of the possibilities and challenges for living qualitative evidence syntheses

**DOI:** 10.1186/s13643-023-02218-0

**Published:** 2023-03-16

**Authors:** Chris Carmona, Christopher Carroll, Susan Baxter

**Affiliations:** School of Health and Related Research (ScHARR), University of Sheffield, Sheffield, UK

**Keywords:** Qualitative evidence synthesis, Health guidelines, Living guidelines, Living systematic review

## Abstract

Over the past decade qualitative evidence synthesis (QES), a range of methods for synthesising qualitative research evidence, has become a valued form of evidence for guideline producers who wish to understand more about patient preference and acceptability of treatments. The surge in interest in living systematic reviews and the appearance of living guidelines as a response to the COVID-19 pandemic potentially weaken the value and usability of QES.

There are currently no published methods for producing living QES, and if QES are to remain of worth to guideline producers then methods for the rapid, frequent updating of them will need to be developed. We discuss some of the similarities and differences between qualitative and quantitative evidence syntheses and highlight areas where development is needed if reviewers are to progress with living approaches to QES.

## Background

Qualitative evidence synthesis (QES) refers to a range of methods for synthesising qualitative research studies and has been in use since the late 1980s [1]. Since 2004, there has been a Cochrane Methods Group tasked with advising the Cochrane Collaboration on policy related to the synthesis of qualitative evidence and the integration of qualitative evidence with Cochrane effectiveness reviews. More recently, QES has become a part of the process of developing evidence-based health guidelines by organizations such as the UK National Institute for Health and Care Excellence (NICE) and by the World Health Organization (WHO) where they have been used by guideline panels to support their decision making [2–4]. This incorporation of QES into health guidelines has been made easier both by methodological developments in the ways that QES are undertaken (for example the introduction of GRADE CERQual [5]), and a drive by guideline-producing organisations to consider the effects of patient preference, feasibility and acceptability on the broader effectiveness of a treatment or intervention when making guideline recommendations [3].

The concept of a living systematic review (LSR) has been in evidence since 2014 [6]. Cochrane defines a LSR as a *“*systematic review which is continually updated, incorporating relevant new evidence as it becomes available” [7]. Prior to the COVID-19 pandemic LSRs were largely theoretical entities, although Cochrane released their first ‘Guidance for the production and publication of Cochrane living systematic reviews’ in 2017 (and updated it in 2019) [8]. During the COVID-pandemic, the need to respond quickly to rapidly changing evidence and practice led to the use of LSRs to inform living guidelines (LG) that could be updated each time the evidence changed in a meaningful way. A living guideline is "an optimisation of the guideline development process to allow updating of individual recommendations as soon as new relevant evidence becomes available" [9].

This surge in interest in LGs has been facilitated by developments in the technologies used to search for and screen evidence using machine learning and also by developments of user-friendly updatable content management systems such as MAGICapp [10]. Living guidelines are also a response to the need for clinicians and healthcare professionals to have access to guidelines based on the best currently available evidence.

The value of producing LGs has been recognised by the Australian National Clinical Evidence Taskforce (NCET), NICE in the UK and the WHO. The WHO has a living guideline for the clinical management of COVID-19 [11], NCET has living guidelines on both COVID-19 [12] and mpox [13], and pillar 2 of the NICE strategy 2021–2026 promises ‘Dynamic, living guideline recommendations’ over the next 3 years [14].

## What are the implications of LGs for the future use of QES in health guideline development?

The increase in focus on LGs based on living systematic reviews poses a challenge to the ‘new era for qualitative research’ described by Lewis and Glenton [15]. Currently, there are no published methods for constant updating of QES or for the development of ‘living QES’. If qualitative methodologists cannot respond to the challenge of developing methods for constantly updating QES, then they run the risk of being side-lined by a focus on quantitative evidence and syntheses for LGs. Qualitative methodologists urgently need to develop methods for making QES ‘living’ in a way that will allow them to be updated alongside LSR.

## Characteristics of living reviews and guidelines

### What makes a systematic review ‘living’?

Cochrane [7] defines a LSR as a systematic review which is continually updated, incorporating relevant new evidence as it becomes available.

They define this in practical terms as LSRs:

- Being underpinned by continual, active monitoring of the evidence (i.e., monthly searches)
- Immediately including any new important evidence (meaning data, studies, or information) that is identified
- Being supported by up-to-date communication about the status of the review and any new evidence being incorporated
- Including pre-defined decisions about how often new evidence is sought and screened and when and how new evidence is incorporated into the review

If the review is set up in the right way from its inception then this can be fairly straightforward. For a review that is authored within a program or application that automatically undertakes analysis, for example, Cochrane’s RevMan [16] or MAGICapp [10], then new data can be added to an existing meta-analysis to generate a new pooled effect estimate, and GRADE (if it is being used) domains for that outcome can be edited if necessary. Lists of included and excluded studies can be updated and new evidence tables inserted.

### What makes a health guideline ‘living’?

It currently seems less clear what the criteria are for a LG. Akl's definition (above) [9] provides a good starting point; however, the processes that need to underpin the guideline's production and maintenance remain exploratory. It is broadly agreed that a process needs to be established whereby new evidence that updates a LSR is assessed in relation to existing guideline recommendations, and then a judgment is made about whether the new evidence is likely to affect the existing recommendation. If it is, then a guideline panel will meet to discuss the evidence and update the recommendation, if not the LSR will be updated but the guideline will stand.

## How can we apply these characteristics to QES?

### Towards a living QES

If we apply the list of criteria from LSRs to QES, one can imagine a scenario where searches for qualitative publications are repeated regularly to identify new studies for inclusion into a QES. Searching for qualitative studies, however, is typically perceived to be more complex than searching for randomised controlled studies [17]. The numbers of records retrieved can be higher, which in turn implies more work for researchers tasked with a frequent sifting of this data. It might be that monthly updates are considered too frequent for most areas of health reviews given that fewer qualitative studies are published [18]. The main challenge for a living QES lies in the ability of the QES to incorporate new evidence quickly and meaningfully in a way that allows decisions to be made by guideline producers about whether a guideline needs updating.

We might suppose that different methods of qualitative evidence synthesis will encounter distinct levels of challenge in attempting to integrate new data into existing themes. It seems likely that the more interpretive approaches to QES, for example, meta-ethnography [1], might require substantial work to incorporate even modest amounts of new data because large parts of the analysis might need to be re-done to take account of the new data. Conversely, one might imagine that for more aggregative approaches to QES, it will be more straightforward to determine the potential impact of new data on existing themes. This might be the case, for example, for aggregative synthesis [19] or approaches that use prespecified frameworks for synthesising data, for example, Best Fit Framework synthesis [20]. If the data match closely to what is already contained in the theme, then the theme may not need updating, other than to add the study details to the review and consider the effects of the study on CERQual decisions (if used) for the theme. Changes to themes might be easily integrated if they require refinement of individual themes or aggregates of codes rather than a wholescale reinterpretation of the data.

There are further considerations that may be unique to QES that need discussion and development, stemming from the very different nature of the evidence used to develop QES. A good example of this is the currency of qualitative data (its up-to-date-ness). While we can probably assume that in the context of an LSR, evidence about the efficacy of a drug is likely to be constant over time, we might not be able to say the same of qualitative data. Prevalent social and individual views and experiences change over time as a society, health care, and health expectations change over time. There might be a requirement for evidence to be removed from a QES as it becomes dated. This raises questions regarding at what point should evidence be ‘retired’ from a living QES? Is there a lifespan for a QES before it becomes incoherent and needs to be completely revised?

### How will such a living QES inform a living health guideline?

In the same way as for a LSR, a guideline producer using a living QES to inform part of a health guideline would need explicit criteria to invest the time and resources necessary to convene a guideline panel to re-examine existing recommendations on the basis of integrating new evidence. There would need to be some belief or expectation that the new evidence would change recommendations. While for quantitative reviews decision-making may be based around whether new evidence changes the effect size (or direction of effect) of an intervention, for qualitative data the implications of adding new studies may be less clear. Qualitative researchers may not be comfortable with the idea of ‘hard’ rules about updating guidelines. Perhaps a meaningful alternative would be to consult a small panel of experts and lay people each time a QES is updated to seek guidance on whether new evidence has the potential to alter existing recommendations.

## Conclusion

The past 10 years have been a period of growth in methods for QES with their value increasingly recognised by organisations that specialise in evidence-based medicine. The emergence of LSRs and LGs requires an urgent response from QES methodologists to develop efficient and effective processes for updating QES quickly and frequently, if the synthesis of evidence from qualitative studies is to meet the needs of health guideline producers.

## Data Availability

Not applicable.

## Abbreviations

COVID-19: Coronavirus disease 2019
GRADE: Grading of Recommendations Assessment, Development and Evaluation
CERQual: Confidence in the Evidence from Reviews of Qualitative Research
LG: Living guideline
LSR: Living systematic review
NCET: Australian National Clinical Evidence Taskforce
NICE: National Institute for Health and Care Excellence (UK)
QES: Qualitative Evidence Synthesis
WHO: World Health Organization
